# Dinoflagellate and ciliate trophic modes database

**DOI:** 10.1128/mra.00154-25

**Published:** 2025-06-10

**Authors:** Erin L. Jones, Susanne Menden-Deuer, Tatiana A. Rynearson

**Affiliations:** 1 Graduate School of Oceanography, University of Rhode Island 54083, Narragansett, Rhode Island, USA; University of California Riverside, Riverside, California, USA

**Keywords:** dinoflagellate, ciliate, trophic ecology

## Abstract

This database synthesizes trophic information on dinoflagellate and ciliate taxa, emphasizing their crucial roles in marine food webs. The compiled database serves as a valuable resource for understanding the ecological importance of these protists across the global ocean.

## ANNOUNCEMENT

Heterotrophic protists play a key role in marine food webs as grazers of primary production, recyclers of organic material, and intermediaries between phytoplankton and higher trophic levels (1–3). Studies over the past decade have expanded our understanding of plankton biodiversity globally (4–7). Heterotrophic protists, especially dinoflagellate and ciliate species, are taxonomically diverse groups of eukaryotic plankton and display a wide range of trophic modes that influence their ecological impact (5, 8). Despite their taxonomic diversity and global prevalence in marine ecosystems, knowledge gaps in taxonomic and trophic reference material have limited our full understanding of heterotrophic protist communities in marine food webs and carbon export pathways. As the taxonomic classification of heterotrophic protists improves, it is critical to connect taxonomy with trophic roles to better capture the ecological importance of such diverse, prevalent, and integral communities.

Over 500 studies and a mixotroph database (9) were reviewed to assign known trophic modes to identified dinoflagellate and ciliate taxa, using multiple literature sources for maximum confidence in classification. Protists were assigned trophic modes (heterotroph, mixotroph, and parasite) based on the lowest possible taxonomic level corresponding to references. Taxonomic synonyms, feeding strategy (e.g., selective herbivore, carnivore, non-constitutive mixotroph, etc.), prey types (bacteria, diatom, chlorophyte, haptophyte, dinoflagellate, copepod, etc.), and equivalent spherical diameter (10) (ESD) ranges were recorded from available references or calculated from organism length (L) and width (W) using the following equation;


$$ESD=2\sqrt{\left(\frac{L\times W}{\pi }\right)}.$$

The trophic modes database (TMDv1.1) is available in a comma-delimited format and in Microsoft Excel (v16.80) to allow for manipulation and sorting. The resulting reference database includes 378 individual taxa from the phyla Dinoflagellata and Ciliophora with trophic classification as heterotroph, mixotroph, or parasite as well as other relevant ecological and taxonomic information.

The trophic modes database provides an accessible resource to reference, characterize, and interpret the functional diversity, ecological roles, and trophic structure of marine protist communities. By developing this database, we have distilled decades of independent research into one accessible reference for future studies.

## Data Availability

This Trophic Modes Database (TMDv1.1) is available in the Zenodo data repository (DOI: 10.5281/zenodo.14866528).
